# Schwannoma of the urinary bladder: a case report

**DOI:** 10.11604/pamj.2014.18.84.4586

**Published:** 2014-05-26

**Authors:** Adil Mazdar, Mohammed Asseban, Hani Aboussalah, Hamad Motia, Hachem Elsayegh, Ail Iken, Lounis Benslimane, Yassine Nouini

**Affiliations:** 1 Department of Urology A, University Hospital IBN SINA, Rabat, Morocco

**Keywords:** Bladder mass, bladder schwannoma, neurofibromatosis, schwannoma

## Abstract

Bladder schwannomas are exceedingly rare, benign or malignant, nerve sheath tumors that are most often discovered in patients with a known diagnosis of Neurofibromatosis type 1 (NF1). A few sporadic case reports of bladder schwannoma have been published in urologic, obstetric/gynecologic, and pathologic journals. We report a case of an isolated schwannoma of the urinary bladder. To our knowledge, this represents only the sixth case of benign schwannoma of the urinary bladder in a patient without von Recklinghausen disease.

## Introduction

Schwannoma of the urinary bladder is an extremely rare tumor. It arises from Schwann cells in nerve sheaths and may be malignant [1] or benign [2] and is often associated with von Recklinghausen′s disease. A few sporadic case reports of bladder schwannoma have been published in urologic, obstetric/gynecologic, and pathologic journals. We report a case of an isolated schwannoma of the urinary bladder in a patient without von Recklinghausen disease.

## Patient and observation

We report the case of a female patient aged 50, with no particular history admitted to our department for management of hematuria.

Her history of the disease was within 3 months of the onset of intermittent total hematuria associated with irritative lower urinary tract signs namely urgency and voiding burns. Clinical examination was normal, apart from hypogastric tenderness.

**Figure 1:**
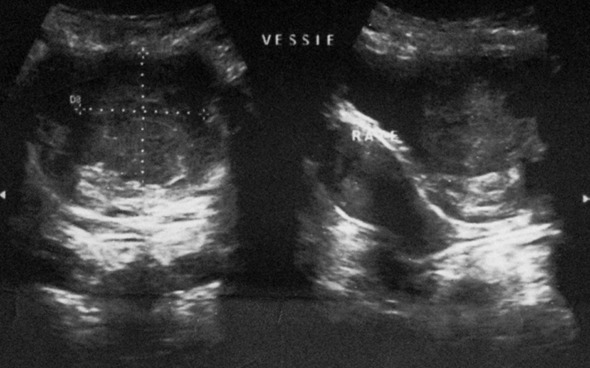
Echographic appearance of schwannoma bladder

Before the clinical abdominopelvic ultrasound was asked a table, she showed a Right and left kidney of normal size, and bladder smooth contour seat was a mass of tissue echo structure (58 * 52 * 15mm) of the postero -lateral right (Figure 1). Laboratory tests were normal, including a urine culture was negative.

**Figure 2:**
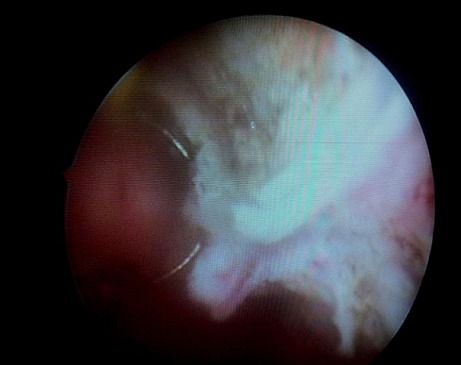
Cystoscopic appearance of the bladder schwannoma: solid tumor of the left lateral side of the bladder

The cystoscopic exploration objectified non- papillary solid tumor of the left bladder trigone and lateral side, both ureteral meatus were free. Biopsies of the lesion were sent to pathology for further evaluation (Figure 2).

**Figure 3:**
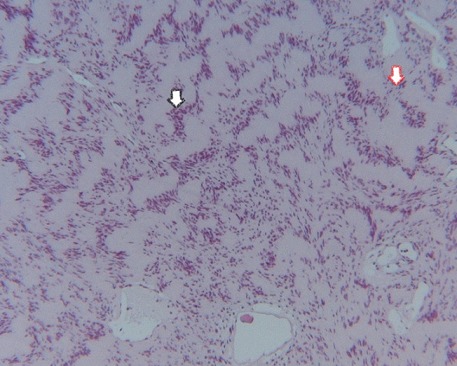
Biphasic tumor: compact hypercellular Antoni A areas (black arrow) and hypocellular Antoni B areas (red arrow) with irregularly spaced vessels. (HE x100)

**Figure 4:**
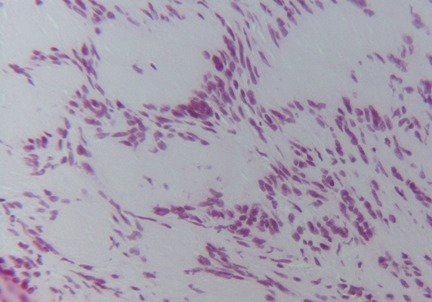
Tumor cells have ill-defined cytoplasm and nuclear palisading. (HE x400)

On macroscopic examination, the submucosal mass appeared tan, smooth and rubbery. The mass was sectioned and stained with Hematoxylin and Eosin (H&E) for further evaluation. Light microscopy revealed a spindle cell neoplasm with areas of hypocellularity (Antoni B) and areas of dense cellularity (Antoni A). Within the densely cellular areas, palisading nuclei alternated with pink, nuclear free zones (Verocay bodies). These findings are highly characteristic of a schwannoma.. Immunohistochemical study showed that tumor cells express the anti PS100 Ac, which confirms the final diagnosis of schwannoma of bladder ( Figure 3, Figure 4).

**Figure 5:**
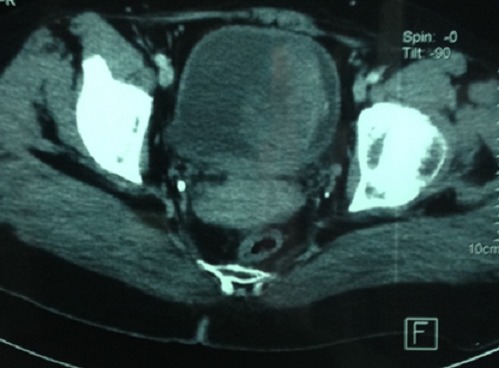
Axial image from a contrast abdominal CT in soft tissue windows demonstrates a homogeneous, well-circumscribed, solid mass on the left anterolateral bladder wall

CT of the abdomen and pelvis was performed documenting no evidence of tumor extension outside the confines of the bladder; the mass was well circumscribed with a density of 32 Hounsfield units (HU) on the left side of the bladder wall. The mass was isodense relatively to the wall of the bladder and measuring 5.4 X 6.1 cm of axial dimensions (Figure 5).

The final treatment consisted of the production of a transurethral resection of bladder with good control of the tumor. The patient had no immediate post-operative complications. She was followed for 5 months without tumor recurrence before moving out of state.

## Discussion

It was in 1910 that VEROCAY described the first case of nerve sheath tumor and gives it the name of neuroma. In 1932, Masson [3] shows that such tumors derived of Schwann cells forming the sheath that surrounds nerve fibers and proposes thus the name of schwannoma. In 1953 STOUT suggests adopting the term of neurilemnoma, which is accepted by most authors, the key is to remember this type of tumor that originates from the perineural sheath and not the nerve fibers themselves [4].

Schwannoma is a ubiquitous lesion may sit in any part of the body where a nerve sheath is present, including the bladder. ROBERT [5] giving the following distribution: members: 53.1% cases, trunk 13%, head and neck: 13.9%. The primary lesion of the bladder is exceedingly rare indeed Brown and Futter [6] in 1987, did find that three cases in the English literature. Indeed it represents < 0.1% of all bladder tumors [1]. Bladder Schwannomas have no gender predilection, are most common in the 4th-6th decade) [1, 7, 8]. These tumors are usually slow growing and benign, although malignant variants have been reported (< 5%) [9]. These schwannomas must be distinguished from neurofibromas of Von Recklinghausen disease whose location bladder is much more common and can be very serious, including in children [10, 11].

Clinical symptoms are nonspecific, most often provide hematuria and irritative lower urinary tract seats. Nevertheless, additional examinations of imaging are needed to advance in the diagnosis [12].

Ultrasound has give a notion of mass is now widely supplanted by scanner (CT) and Magnetic resonance imaging (MRI). Both tests give a clear idea of the location of the tumor [13]. The CT appearance of a schwannoma is non-specific. However, a few characteristic features exist. Bladder schwannomas appear isodense or hypodense to surrounding muscle [14]. Schwannomas are not typically associated with calcifications [8]. Their enhancement tends to be dense and homogeneous when small and increasingly heterogeneous as they enlarge [15]. However, enhancement patterns have not been shown to be a reliable way to differentiate schwannomas from other bladder tumors [16]. Despite the inability to adequately differentiate various bladder tumors, contrast-enhanced CT is excellent in detecting up to 97% of all bladder neoplasms [17]. MRI is slightly more sensitive than CT for the evaluation of suspected schwannomas, but differentiation between a bladder schwannoma and carcinoma remains difficult. Both schwannomas and carcinomas are usually isointense to skeletal muscle on T1 weighted imaging (T1WI) and isointense to slightly hyperintense to skeletal muscle on T2 weighted imaging (T2WI) [14, 18]. Given their similar appearance on both CT and MRI, clinical history is of the upmost importance.

The diagnosis can only be made by histology. Two highly characteristic patterns for schwannoma can be seen on H&E staining. Antoni A areas consist of compact intersecting spindle cells with elongated nuclei arranged in parallel bundles, incomplete whorls and complete whorls (Verocay bodies) [19]. Antoni B areas are composed of loosely arranged spindle cells [19]. Positive S100 immunohistochemistry is the pathognomonic pathology finding of a schwannoma [20].

Treatment of these tumors has included cystectomy, transurethral resection, observation, radiotherapy, chemotherapy, and urinary diversion. The current belief regarding treatment is that in the absence of other sequelae, these neural sheath tumors can be treated conservatively [7].

The prognosis is related to the risk of recurrence, which is extremely rare, if the exeresis is complete. The Benin schwannoma rarely escalates. One case has been described [21] or malignant schwannoma appeared remotely on site excision of a schwannoma Benin. The same low risk transformation and recurrence makes postoperative monitoring annual CT necessary.

## Conclusion

Bladder schwannoma is a very rare tumor whose diagnosis is histological. Treatment of this tumor remains uncodified, but a full excision of the tumor is mandatory to avoid recurrence. Although the degeneration of the tumor is exceptional surveillance by an annual scanner is legitimate.

## References

[1] Rober PE, Smith JB, Sakr W, Pierce JM (1991). Malignant peripheral nerve sheath tumor (malignant schwannoma) of urinary bladder in von Recklinghausen neurofibromatosis. Urology..

[2] Winfield HN, Catalona WJ (1985). An isolated plexiform neurofibroma of the bladder. J Urol..

[3] Masson P (1932). Tumors of the peripheral nerves. Am J Cancer..

[4] Singer AJ, Anders KH (1996). Neurilemoma of the kidney. Urology..

[5] Robert R (1977). Les tumeurs nerveuses primitives tronculaires des membres: à propos de 9 cas, Thèse médecine. Nantes.

[6] Brown IR, Futter NG (1997). Primary neurilemnoma of the bladder. Br J Urol..

[7] Cummings JM, Wehry MA, Parra RO, Levy BK (1998). Schwannoma of the Urinary Bladder: A Case Report. International Journal of Urology..

[8] Gafson I, Rosenbaum T, Kubba F, Meis JM, Gordon AD (2008). Schwannoma of the bladder: A rare pelvic tumour. Journal of Obstetrics and Gynecology..

[9] Eltoum IA, Moore RJ, Cook W, Crowe DR, Rodgers WH, Siegal GP (1999). Epithelioid Variant of Malignant Peripheral Nerve Sheath Tumor (Malignant Schwannoma) of the Urinary Bladder. Annals of Diagnostic Pathology..

[10] Messina AM, Strauss RG (1976). Pelvic neurifibromatosis. Obst Gynecol..

[11] Nguyen HT, Kogan BA, Hricak H, Turzan C (1997). Plexifor neurofibroma involving the genitourinary tract in children: case reports and review of the literature. Urology..

[12] Fukui S, Kiba K, Shinohara M, Yoneda T (2010). Schwannoma arising from the urinary bladder wall: a case report. Hinyokika Kiyo..

[13] Fernandez JM, Val-Bernal JF, Escaf S, Sahagun JL (1997). Retrovesical cellular schwannoma. Br J Urol..

[14] Murovic JA, Kim DH, Kline DG (2006). Neurofibromatosis-associated nerve sheath tumor-case report and review of the literature. Neurosurgical focus..

[15] Wong-You-Cheong JJ, Woodward PJ, Manning MA, Sesterhenn IA (2006). Neoplasms of the urinary bladder: radiologic-pathologic correlation. Radiographics..

[16] Merkle EM, Wunderlich A, Aschoff AJ, Rilinger N (1998). Virtual cystoscopy based on helical CT scan datasets: perspectives and limitations. Br J Radiol..

[17] Kim JK, Park SY, Ahn HJ, Kim CS, Cho KS (2004). Bladder Cancer: Analysis of Multi-Detector Row Helical CT Enhancement Pattern and Accuracy in Tumor Detection and Perivesical Staging. Radiology..

[18] Murphey MD, Smith WS, Smith SE, Kransdorf MJ, Temple HT (1999). Imaging of musculoskeletal neurogenic tumors: radiologic-pathologic correlation. Radiographics..

[19] Boning L, Gengxi S, Bo Z, Kangfu L, Ianming W, Kunhao F (2004). Neuroradiological findings of intracranial schwannomas not arising from the stems of cranial nerves. Br J Radiol..

[20] Wang W, Montgomery E, Epstein J (2008). Benign Nerve Sheath Tumors on Urinary Bladder Biopsy. American Journal of Surgical Pathology..

[21] Das Gupta TK, Brasfiels RD, Strong EW, Hajolu SZ (1969). Benign solitary schwannoma. Cancer..

